# Prediction of Grain Structure and Texture in Twin-Roll Cast Aluminum Alloys Using Cellular Automaton–Finite Element Method

**DOI:** 10.3390/ma18051075

**Published:** 2025-02-27

**Authors:** Han-Gyoung Cho, Young Do Kim, Min-Seok Kim

**Affiliations:** Department of Materials Science and Engineering, Hanyang University, 222, Wangsimni-ro, Seongdong-gu, Seoul 04763, Republic of Korea

**Keywords:** CA-FE model, twin-roll casting, Al-Zn-Mg-Cu alloy, pure aluminum, grain structure, texture

## Abstract

The twin-roll casting (TRC) process has gained significant attention for aluminum sheet production due to its cost-effectiveness and high processing efficiency. However, controlling the initial grain structure of TRC strips remains challenging due to the absence of a hot rolling stage, necessitating an advanced predictive modeling approach. In this study, a cellular automaton–finite element (CA-FE) model was developed to predict the grain structure and texture of aluminum strips fabricated via TRC. Both pure Al and AA7075 alloys were cast under identical conditions using a pilot-scale horizontal twin-roll caster, and their microstructures were characterized experimentally. The developed model incorporated a Gaussian nucleation distribution function and an equivalent binary approach to account for the solidification behavior of multicomponent alloys. The CA-FE simulation results successfully reproduced the key aspects of solidification, grain structure, and texture evolution of TRC strips. The predicted temperature distribution and solid fraction evolution showed distinct differences between the alloys, with pure Al forming columnar grains and AA7075 developing a fully equiaxed structure, which closely matched the experimental findings. Additionally, texture analysis using inverse pole figures (IPFs) and pole figures (PFs) revealed a clear <001> orientation in pure Al, whereas AA7075 exhibited a random texture, both of which were well captured by the CA-FE model. The findings indicate that the developed model offers a reliable prediction of the solidification microstructure and texture evolution in TRC strips, making it a valuable tool for optimizing continuous casting processes.

## 1. Introduction

In recent years, the twin-roll casting (TRC) process has gained substantial attention in the aluminum sheet industry due to its advantages of low equipment and processing costs [1]. This process allows for the production of strips with a thickness ranging from approximately 3 to 12 mm directly from molten metal, eliminating the need for surface scalping and hot rolling processes, which are essential in conventional slab-based manufacturing [2]. Despite these advantages, industrial-scale TRC production has been limited to low-alloy systems, such as the 1xxx and 3xxx series [2]. To broaden the application of aluminum sheets in transportation and other advanced fields, ongoing research and development efforts are focused on enabling the production of high-strength aluminum sheets, including 5xxx, 6xxx, and 7xxx series alloys [3,4].

The initial grain size and structure of as-cast strips are critical factors in determining the microstructure of subsequent cold-rolled products [5]. For strips produced via the TRC process, the absence of a hot rolling stage significantly reduces opportunities for grain structure control compared to conventional slab-to-hot rolling processes. Therefore, the precise controlling of the initial grain structure is essential for optimizing the microstructure and mechanical properties of final sheets [6].

Aluminum alloys exhibit a wide range of grain structures, influenced by alloy series, composition, and casting conditions. In the TRC process, one of the most effective approaches to microstructure control is the utilization of simulation technologies. Recent advancements in solidification simulation models and the development of cellular automaton–finite element (CA-FE) algorithms have significantly improved the ability to predict grain structures [7,8,9]. Rappaz and Gandin developed a CA-based solidification model that effectively predicts macroscopic grain structures [7,8]. Their model accounts for grain position, crystallographic orientation, and the growth kinetics of the dendrite tip, enabling a more accurate representation of the solidification process. A key advancement of their approach is the integration of the CA algorithm with finite element (FE) heat transfer calculations, which allows the model to be applied under non-uniform thermal conditions, enhancing its applicability to complex solidification phenomena. Numerous researchers have utilized the CA-FE model to predict solidification grain structures in conventional casting and welding processes [10,11,12,13,14,15,16,17,18].

While CA-FE models have primarily been applied to conventional solidification processes, recent research has explored their adaptation to more complex continuous casting methods [19,20]. Among these, research and development in TRC for aluminum alloys remain relatively underdeveloped. Although some studies have utilized CA-FE models to predict microstructures in materials such as steel [19] and Ti alloys [21], their application to aluminum alloys in TRC remains largely unexplored. Previous studies have predicted the rolling texture in pure Al strips fabricated under traditional low-speed/high-load conditions [22]. However, research on the prediction of grain structure and texture evolution in various aluminum alloys processed via TRC is still highly limited. Although predicting deformation texture using CA-FE models is inherently challenging, recent advancements in TRC technology have led to higher casting speeds and significant changes in the as-cast microstructure, shifting from a rolling texture to a more distinct cast microstructure [23]. These developments offer a valuable opportunity to apply CA-FE modeling in TRC processes.

In CA-FE models, heterogeneous nucleation is described using a continuous nucleation distribution function, which assumes that nucleation sites follow a Gaussian distribution [7]. This approach relies on three key parameters: maximum nucleation density, mean nucleation undercooling, and the standard deviation of nucleation undercooling. Since nucleation occurs both at the mold surface and in the bulk liquid during the casting process, a total of six parameters is required. These parameters are influenced by various process conditions and have a direct impact on the accuracy of model predictions, making their careful selection essential [24]. However, obtaining generalized parameter values through experiments is particularly challenging [25]. Therefore, to effectively apply the model to continuous casting processes, a thorough understanding of the correlation between process variables and nucleation-related parameters is crucial.

The macroscopic grain structures formed during the solidification of aluminum castings are generally classified into two types: columnar and equiaxed grain structures [26]. Depending on the casting conditions, either a single-grain structure may develop or a mixed structure consisting of both types may appear [20]. In the TRC process, which operates under relatively low melt superheat conditions compared to conventional casting processes, the grain structure of aluminum strips exhibits significant variations [27]. Pure aluminum tends to form a columnar grain structure, while high-alloy systems such as 5xxx and 7xxx series alloys predominantly develop equiaxed grain structures [28]. In this study, we aim to develop a simulation model capable of predicting grain structures and texture evolution in pure aluminum and high-strength Al-Zn-Mg-Cu alloys. To achieve this, the grain structure, grain size, and texture of experimentally fabricated TRC strips will be analyzed and compared with the simulation results. Through this comparative analysis, we demonstrate the feasibility of microstructure prediction modeling using the CA-FE model and provide fundamental data for advancing microstructure prediction in aluminum alloys.

## 2. Materials and Methods

### 2.1. TRC Process for Strip Fabrication

**Figure 1:**
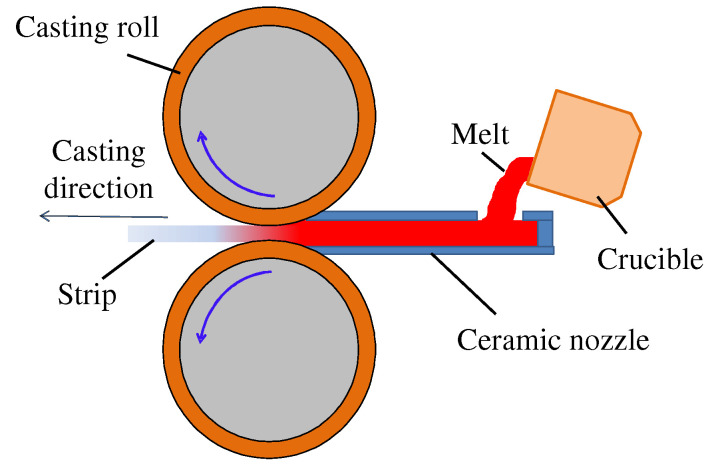
Schematic diagram of the pilot-scale TRC process.

To obtain a diverse range of grain structures, commercially available pure Al and AA7075 aluminum alloys were used to produce TRC strips. The compositions of the alloys used in this study are summarized in Table 1. Each alloy was melted in an electric furnace, followed by degassing with Ar gas for approximately 10 min before TRC. The chemical compositions of the as-cast strips were analyzed using the ICP-OES (inductively coupled plasma optical emission spectrometry) method. Figure 1 shows a schematic diagram of the pilot-scale TRC process employed in this study. The strips were fabricated using a horizontal twin-roll caster equipped with two casting rolls, each with a diameter of 300 mm. The roll surfaces were made of Cu shells. Both alloys were cast under the same 4 m/min speed condition, and the other casting parameters are summarized in Table 2. During the TRC process, the roll load was minimized as much as possible while maintaining strip quality to minimize the rolling effect during strip production. The resultant strip thicknesses of pure Al and AA7075 alloy were 3.6 mm and 4.4 mm, respectively. For the TRC strips, the longitudinal cross-sections were mechanically polished and anodized at 40 V in a 3.3% solution of HBF4 in distilled water to reveal the grain structure. The strip textures were observed by electron backscattering diffraction (EBSD) using a TESCAN MIRAII FE-SEM (Brno, Czech Republic) equipped with a Hikari EBSD detector at an accelerating voltage of 20 kV. The EBSD results were analyzed using OIM analysis 7 software provided by TSL, Co., Ltd., New Taipei City, Taiwan.

### 2.2. Simulation Model for Twin-Roll Casting Process

#### 2.2.1. TRC Model Geometry and Boundary Condition

**Figure 2:**
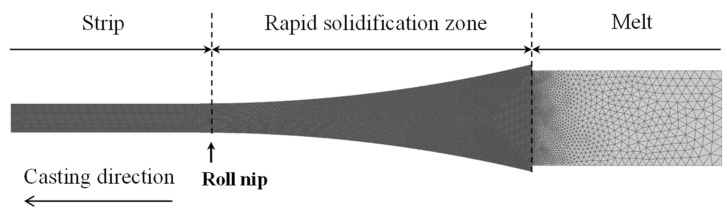
Geometry and mech of the TRC simulation model.

Finite-element simulations of the TRC process were performed using the commercial software ProCAST (ver. 2021). Figure 2 illustrates the geometry and mesh structure of the TRC simulation model. The mesh size was adapted based on the importance of analysis in each region, with a 0.1 mm mesh applied to regions experiencing rapid solidification to ensure higher calculation accuracy. The boundary conditions for the heat transfer analysis are summarized in Table 3. The interfacial heat transfer coefficient (IHTC) at the roll/melt interface (*h_roll_*_/*melt*_), which directly influences solidification behavior, was calculated using the following empirical equation derived from previous studies [29,30].

*h* = 44.8*v*^0.55^ (1)

where *h* is the roll/melt IHTC (kW/m^2^K) and *v* is the roll rotation speed (m/s).

**Figure 3:**
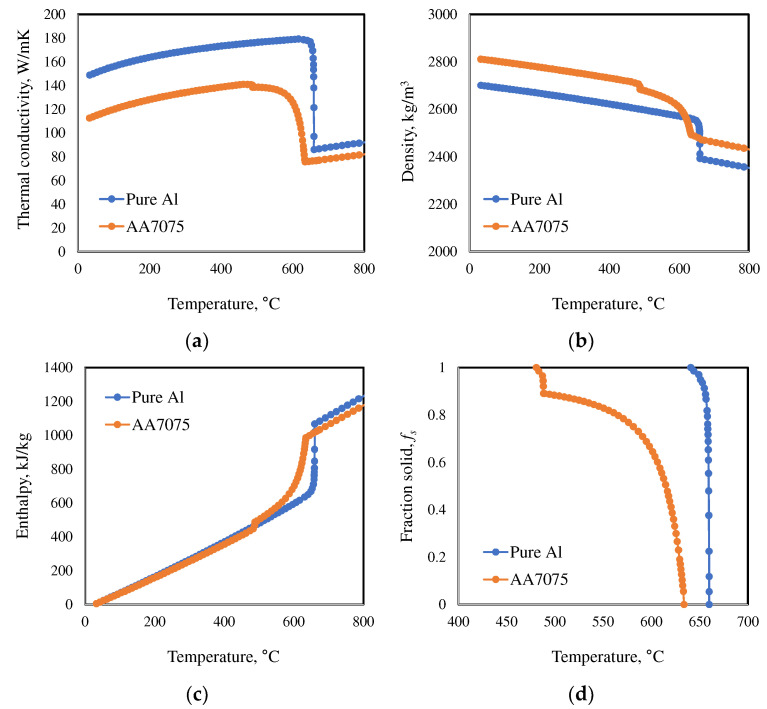
Thermo-physical properties of aluminum alloys used in the simulation: (**a**) conductivity; (**b**) density; (**c**) enthalpy; (**d**) fraction solid vs. temperature.

The thermo-physical properties of the alloys were obtained from the PanAl2021 database included with the ProCAST software (Figure 3). The alloy properties were calculated under Scheil cooling conditions, taking into account the high cooling rates of the TRC process.

#### 2.2.2. Cellular Automaton (CA) Model

The CA-FE model was employed to predict the solidification grain structure and texture of TRC strips. To calculate the final grain structure, computations must account for both nucleation and growth mechanisms. In this model, nucleation sites are assumed to follow a Gaussian distribution, which is used to calculate heterogeneous nucleation occurring at both the surface of the casting rolls and within the melt [31]. The grain density as a function of undercooling (∆*T*) can be expressed as follows:

(2) $$\frac{dn}{d(∆T)}=\frac{n_{max}}{∆T_{\sigma }\sqrt{2\pi }}exp\left[\frac{{\left(∆T-\bar{∆T}\right)}^{2}}{{2∆T}_{\sigma }^{2}}\right]\;$$

where *n_max_* is the maximum nucleation density, $\bar{∆T}$ is the mean nucleation undercooling, and $∆T_{\sigma }$ is the standard deviation of nucleation undercooling. Then, the total nucleation density as a function of undercooling can be defined as follows:

(3) $$n\left(∆T\right)=\int_{0}^{∆T}\frac{dn}{d\left(∆T\right)}d(∆T)\;$$

The Kurz–Giovanola–Trivedi (KGT) model was utilized to describe the growth kinetics of dendrite tips [32]. However, as this model was originally developed for binary alloy systems, an equivalent binary approach was adopted to apply it to multicomponent alloys such as Al-Zn-Mg-Cu-based alloys [33]. The equivalent binary value can be calculated as follows [24]:

(4) $$\bar{C_{0}}=\sum C_{0,i}\;$$

(5) $$\bar{m}=\frac{\sum m_{i}\cdot C_{0}}{\bar{C_{0}}}\;$$

(6) $$\bar{k}=\frac{\sum k_{i}\cdot m_{i}\cdot C_{0,i}}{\bar{m}\cdot \bar{C_{0}}}\;$$

where *C*_0_ is the initial composition of the alloy, m is the slope of the Al-X liquidus, and *k* is the partition coefficient, as listed in Table 4.

In conventional casting processes, the total undercooling at the dendrite tip can be obtained as the sum of four contributions as follows [7]:

(7) $$∆T=∆T_{C}+∆T_{t}+∆T_{k}+∆T_{r}$$

where $∆T_{C}$ is the undercooling contributions of solute diffusion, $∆T_{t}$ is the thermal diffusion, $∆T_{k}$ is the attachment kinetics, and $∆T_{r}$ is the liquid/solid interface curvature. For most metallic alloys, solute undercooling dominates, while the contributions from the other factors are relatively small. The growth rate of the dendrite tip was calculated using the following relationship [20]:

(8) $$v\left(∆T\right)=a_{2}∆T^{2}+a_{3}∆T^{3}$$

where a_2_ and a_3_ are the dynamic coefficients of dendrite growth. In this study, a_2_ = 0.030 m/s∙K^2^ and a_3_ = 0.003 m/s∙K^2^ for the pure Al, and a_2_ = 0 m/s∙K^2^ and a_3_ = 1.577 × 10^−6^ m/s∙K^2^ for the AA7075 alloy were used.

## 3. Results and Discussion

### 3.1. Solidification Behavior in Twin-Roll Cast Strips

**Figure 4:**
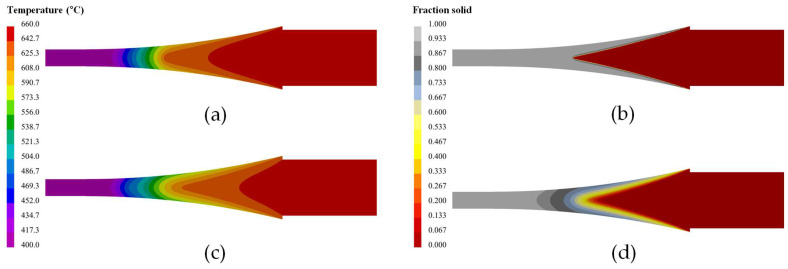
FE simulation results: (**a**) temperature distribution and (**b**) solid fraction distribution of the pure Al strip; (**c**) temperature distribution and (**d**) solid fraction distribution of the AA7075 strip.

Figure 4 presents the predicted temperature distribution and solidification behavior (solid fraction distribution) during the production of pure Al and AA7075 alloys using the TRC process. As shown in the figure, despite being cast under the same casting conditions, the two alloys exhibited distinctly different solidification behaviors, primarily due to their respective solidification temperature ranges, as illustrated in Figure 3d. For pure Al, which has an almost negligible solidification temperature range, the solidification occurred with a well-defined planar growth front at the roll surface as the solidification shell formed. In contrast, the AA7075 alloy, with a wide solidification temperature range, exhibited mushy growth in the semi-solid state as the solidification shell developed. These results highlighted the influence of alloy composition on the solidification mechanism in the TRC process. Using the developed FE model, the grain structure of both alloys was predicted to demonstrate a strong agreement with the experimentally observed solidification behaviors.

### 3.2. Prediction of Solidification Grain Structure in TRC Strips

**Figure 5:**
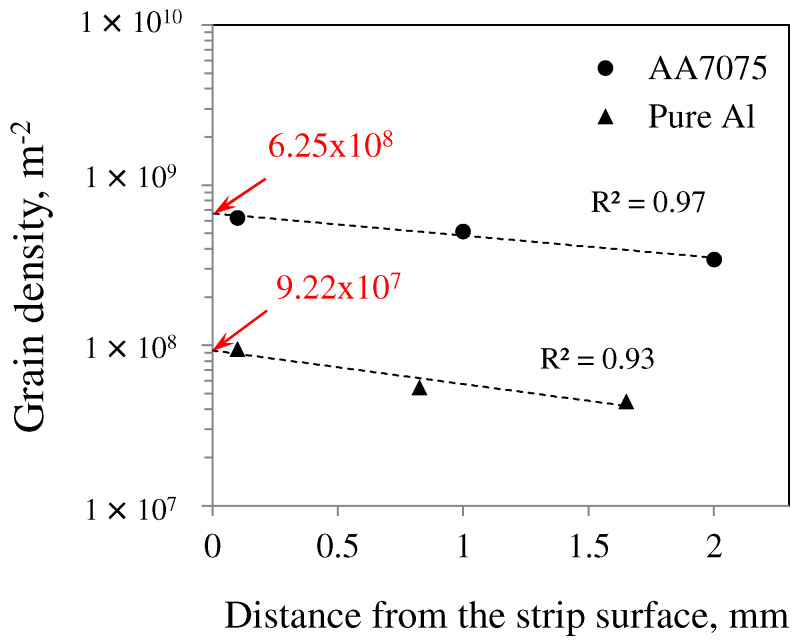
The grain density distribution in the pure Al and AA7075 alloy strips.

As summarized in Table 5, the CA-FE model requires six parameters related to surface and volume nucleation for grain nucleation calculations. To determine maximum surface grain density, the grain sizes were experimentally measured in various regions of the TRC strips, and the grain density per unit area was calculated. Figure 5 shows the grain density distribution across various regions of the pure Al and AA7075 alloy strips. Due to the difficulty of experimentally measuring grain sizes at the strip surface directly in contact with the casting rolls, measurements were instead conducted at three locations: approximately 100 μm beneath the surface, at the middle region (about one-quarter of the strip thickness from the surface), and at the strip center. Using these experimental results, the maximum surface grain density for each alloy was estimated. The predicted maximum surface grain densities for pure Al and AA7075 alloy strips were approximately 9.22 × 10^7^ m^−^^2^ and 6.25 × 10^8^ m^−^^3^, respectively. Previous studies have shown that the mean nucleation undercooling ($\bar{∆T_{S}}$), and the standard deviation of undercooling in surface nucleation, (∆T_σ,S_) have minimal influence on grain structure predictions. Therefore, in this study, fixed values of $\bar{∆T_{S}}$ = 1.0 K, $∆T_{\sigma ,S}$ = 0.1 K, $∆T_{\sigma ,V}$ = 1.0 K were used. Since bulk nucleation data ($\bar{∆T_{V}}$ and $n_{max,V}$) are difficult to obtain experimentally, these values were determined iteratively by adjusting them until the simulation results reasonably matched the experimental observations.

**Figure 6:**
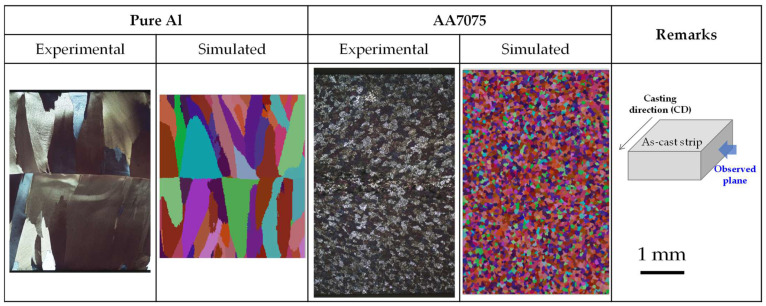
Experimentally observed and simulated grain structure of TRC strips.

**Figure 7:**
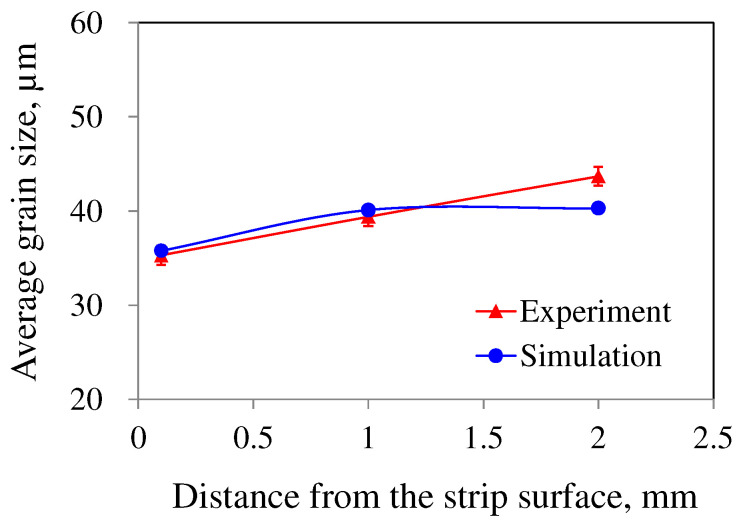
Experimental and simulation results of grain size variation across various positions in the AA7075 strip.

Figure 6 summarizes the macroscopic grain structure observations and simulation results of pure Al and AA7075 strips produced via the TRC process. For pure Al, coarse columnar grains were well-developed perpendicular to the strip surface. In contrast, the AA7075 alloy exhibited relatively fine equiaxed grains distributed throughout the strip, with no columnar grain region. The simulation results, calculated using the parameters in Table 5, closely matched the experimentally observed grain structures in both alloys. Figure 7 presents a comparison between the experimental and simulated grain sizes across various regions of AA7075 strips, as also shown in Figure 6. The grain size increased from the strip surface to the center, and the simulation results predicted the experimental grain size quite accurately.

**Figure 8:**
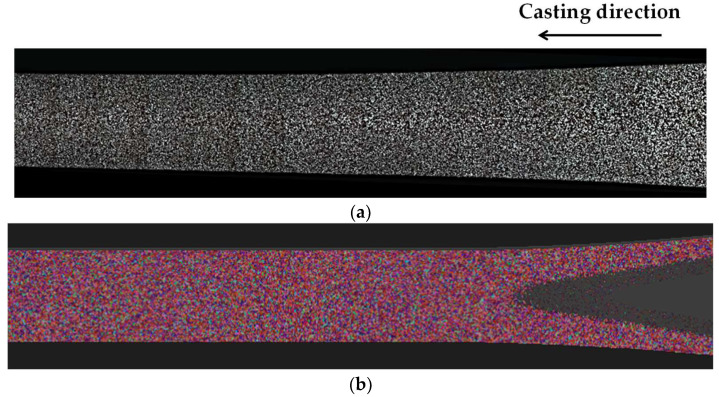
(**a**) Grain structure of the longitudinal cross-section of the experimentally interrupted AA7075 strip and (**b**) corresponding simulation result.

The applicability of the CA-FE model developed in this study was evaluated across a broad range of regions in the AA7075 strip. Figure 8 shows the cross-sectional grain structure of a strip obtained from an intentionally interrupted TRC experiment. Figure 8a provides an extensive view, covering the region from between the rolls to the area where the strip exits the caster. When the CA-FE model developed in this study was applied, it accurately predicted the grain structure and grain size throughout the strip, from the initial solidifying shells to the fully solidified region (Figure 8b).

### 3.3. Prediction of Texture in TRC Strip

**Figure 9:**
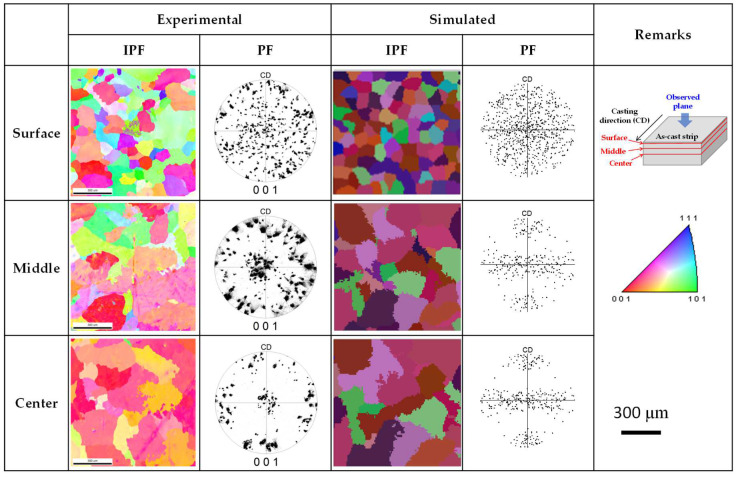
Experimental and simulation results of grain orientation (inverse pole figure) and pole figure (PF) across various regions of the pure Al strip.

The grain textures across various regions of the pure Al and AA7075 strips were predicted using the CA-FE model. Figure 9 shows the grain orientation map and texture of the pure Al strip based on inverse pole figures (IPFs) and {001} pole figures (PFs). To investigate changes in the grain orientation along the depth direction from the strip surface, experiments were conducted in three specific regions as indicated in the annotations of Figure 9. The experimental results in Figure 9 showed that the near-surface region exhibited relatively fine grains and a random texture with no specific orientation. This region corresponds to the chill zone of typical casting microstructures, where extensive nucleation and growth occur on the casting mold [34]. Due to the disordered competition among numerous grains, no distinct orientation was observed in this area. Moving toward the interior of the strip, the grain size increased, and a clear <001> orientation developed, indicating directional grain growth. This phenomenon aligns with the typical growth direction of aluminum dendrites, where dendrites predominantly grow in the <001> direction [35], opposite to the heat flow direction, i.e., perpendicular to the surface of the casting roll. The simulation results in Figure 9 demonstrated that the CA-FE model accurately predicted trends in grain structure, size, and texture variations, showing strong agreement with the experimental observations.

**Figure 10:**
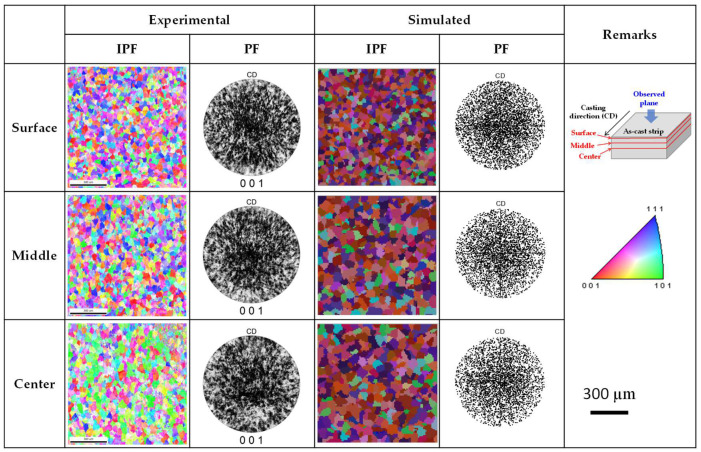
Experimental and simulation results of grain orientation (inverse pole figure) and pole figure (PFs) across various regions of the AA7075 strip.

Figure 10 illustrates the changes in grain structure and texture across various regions of the AA7075 strip. The experimental results revealed that the strip exhibited fine equiaxed grains throughout its cross-section, with a random texture observed across all regions. Such equiaxed grain structures are commonly observed during the production of high-alloy aluminum alloys using the TRC process [6,28]. This phenomenon primarily results from the continuous casting being conducted under relatively low-temperature conditions (i.e., low melt superheat), compared to conventional casting processes, which strongly influence the as-cast microstructure [28]. In Figure 10, the simulation results accurately predicted these grain structure and texture characteristics, demonstrating good agreement with the experimental findings. As demonstrated in this study, the application of the CA-FE model in continuous casting processes enables the reliable prediction of the initial microstructure of the as-cast ingots. This model is not only applicable to the TRC process but also to conventional direct-chill casting processes [20]. The implementation of such models in various ingot production processes is expected to enhance process efficiency, reduce costs, and improve overall microstructure control, making them valuable tools for industrial applications.

The applicability of the CA-FE models to complex continuous casting processes such as TRC was demonstrated in this study. The current FE model employs a fixed interfacial heat transfer coefficient (IHTC) to predict solidification behavior, which has been validated based on previous studies. However, further research is needed to refine the dynamic representation of IHTC, considering variations in process conditions that may influence heat transfer efficiency. Improving the accuracy of heat transfer modeling will contribute to more precise solidification behavior predictions. Additionally, while this study focused on the microstructural prediction of pure Al and AA7075 alloys, the developed model has the potential to be extended to other aluminum alloy systems, such as 5xxx and 6xxx series. Future studies should incorporate experimental validation for a broader range of alloys to enhance the general applicability of the model.

## 4. Conclusions

In this study, a finite element (FE) model was developed to analyze the solidification behavior of aluminum strips during twin-roll casting (TRC). The FE model successfully predicted the temperature distribution and solid fraction evolution, providing a foundation for understanding the solidification process. The simulation results revealed significant differences in the solidification mechanisms of the two alloys, primarily influenced by their solidification temperature ranges. Specifically, pure Al exhibited planar solid growth, forming a well-defined solidification front at the roll surface, whereas AA7075 alloy underwent mushy-type solidification, characterized by a gradual transition in the semi-solid region due to its wider solidification temperature range. Based on the FE model, a cellular automaton–finite element (CA-FE) model was introduced to predict the solidification grain structure and texture evolution. The model incorporated a Gaussian nucleation distribution function and an equivalent binary approach to simulate multicomponent alloy solidification behavior. Experimental validation using pure Al and AA7075 strips demonstrated that the model accurately captured the distinct microstructural features of each alloy—pure Al exhibited coarse columnar grains with a strong <001> orientation, whereas AA7075 developed fully equiaxed grains with a random texture. The grain size predictions showed strong agreement with experimental observations across various strip regions, confirming their reliability in microstructure prediction. Although this study simplifies certain aspects of the casting process, such as the fixed IHTC and nucleation parameters, these assumptions were validated based on previous research and were found to be sufficient for predicting macroscopic microstructures. Future work can focus on refining these parameters dynamically and extending validation to a wider range of aluminum alloys.

## Figures and Tables

**Table 1:** Chemical composition of the alloys fabricated via the TRC process. (wt.%).

Alloy		Zn	Mg	Cu	Cr	Si	Fe	Ti	Al
Pure Al	target	-	-	-	-	-	-	-	Bal.
	ICP	-	-	-	-	0.04	0.1	-	Bal.
AA7075	target	5.5	2.5	1.5	0.2	-	-	-	Bal.
	ICP	5.18	2.27	1.49	0.22	0.11	0.23	0.05	Bal.

**Table 2:** Process parameters for TRC.

Casting Condition	Value
Melt temperature	680 °C
Casting speed	4 m/min
Set-back distance	35 mm
Strip width	100 mm

**Table 3:** The initial and boundary conditions for LPDC.

Condition	Value
Melt temperature	680 °C
Ambient temperature	25 °C
Roll temperature	50 °C
Strip/air IHTC, *h_strip_*_/*air*_	12 W/m^2^K
Roll/melt IHTC, *h_roll_*_/*melt*_	8600 W/m^2^K
Nozzle/melt IHTC, *h_nozzle_*_/*melt*_	adiabatic

**Table 4:** Pseudo-binary phase diagram data for the pure Al and AA7075 alloy.

Alloy	Element	*C*_0,i_ (wt.%)	*m*_i_ (K/wt.%)	*k*_i_
Pure Al	Si	0.04	−5.80	0.11
	Fe	0.1	−3.22	0.02
AA7075	Cr	0.22	10.09	4.59
	Cu	1.49	−2.59	0.11
	Fe	0.23	−4.51	0.03
	Mg	2.27	−4.65	0.30
	Si	0.11	−6.55	0.09
	Ti	0.05	42.20	13.00
	Zn	5.18	−1.87	0.32

**Table 5:** Nucleation parameters for CA-FE simulation of the TRC process.

Condition	Surface Nucleation			Volume Nucleation		
	$\bar{∆T_{S}}$ (K)	${∆T}_{\sigma ,S}$ (K)	$n_{max,S}$ (m^−2^)	$\bar{∆T_{V}}$ (K)	${∆T}_{\sigma ,V}$ (K)	$n_{max,V}$ (m^−3^)
Pure Al	1.0	0.1	9.22 × 10^7^	0.5	1.0	9.50 × 10^8^
AA7075	1.0	0.1	6.25 × 10^8^	5	1.0	2.60 × 10^13^

## Data Availability

The original contributions presented in the study are included in the article, further inquiries can be directed to the corresponding author.
